# Association between radiographic hand osteoarthritis and bone microarchitecture in a population-based sample

**DOI:** 10.1186/s13075-022-02907-6

**Published:** 2022-09-17

**Authors:** Canchen Ma, Dawn Aitken, Feitong Wu, Kathryn Squibb, Flavia Cicuttini, Graeme Jones

**Affiliations:** 1 Menzies Institute for Medical Research, University of Tasmania, 17 Liverpool Street, Hobart, TAS 7000 Australia; 2 Department of Epidemiology and Preventive Medicine, Monash University Medical School, Melbourne, VIC 3181 Australia

**Keywords:** Bone size, Bone mineral density, HRpQCT, Radiography, Hand osteoarthritis

## Abstract

**Background:**

Subchondral bone plays an important role in the pathogenesis of radiographic osteoarthritis (OA). However, the bony changes that occur in hand OA (HOA) are much less understood. This study aimed to describe the association between radiographic HOA and high-resolution peripheral quantitative computed tomography (HRpQCT) measures of the hand and radius in a population-based sample.

**Methods:**

A total of 201 participants (mean age 72, 46% female) from the Tasmanian Older Adult Cohort (TASOAC) study underwent HRpQCT assessment of the 2nd distal and proximal interphalangeal (DIP, PIP), 1st carpometacarpal (CMC) joint, and distal radius. Radiographic HOA was assessed at the 2nd DIP, PIP joints, and the 1st CMC joint using the OARSI atlas.

**Results:**

Proximal osteophyte and joint space narrowing (JSN) scores were consistently more strongly associated with HRpQCT measures compared to the distal site with positive associations for indices of bone size (total and trabecular bone area and cortical perimeter but inconsistent for cortical area) and negative associations for volumetric bone mineral density (vBMD). There was a decrease in trabecular number and bone volume fraction with increasing osteophyte and JSN score as well as an increase in trabecular separation and inhomogeneity. Osteophyte and JSN scores in the hand were not associated with HRpQCT measures at the distal radius.

**Conclusions:**

This hypothesis generating data suggests that bone size and trabecular disorganization increase with both osteophyte formation and JSN (proximal more than distal), while local vBMD decreases. This process appears to be primarily at the site of pathology rather than nearby unaffected bone.

**Supplementary Information:**

The online version contains supplementary material available at 10.1186/s13075-022-02907-6.

## Introduction

Osteoarthritis (OA) and osteoporosis are two important and prevalent musculoskeletal conditions. It has long been recognized that the two conditions are associated with each other although the direction of this association is confusing.

It is well known that distinct bony changes occur in knee OA. In post mortem studies of early knee OA, bone remodeling results in rod- to plate-like changes in the trabeculae of the subchondral cancellous bone which is the opposite to the changes of normal aging [1]. Microarchitectural changes in subchondral cancellous tibial bone in OA also include an increase in thickness of the subchondral plate, a decrease in separation of the trabeculae, a decrease in bone marrow spacing, and an increased bone volume fraction [1, 2]. Bone proliferation in the subchondral region results in eburnation and at the bony margins produces osteophytes [3]. The turnover of subchondral bone has been reported to be increased in the early of hip OA, and subchondral bone properties are similar between osteoporosis and the early hip OA [4, 5].

The bony changes that occur in hand OA is much less understood. Etiologically, it is proposed that in early hand osteoarthritis (HOA), microarchitectural changes take place in the subchondral trabecular bone [6]. Advanced technology such as high-resolution peripheral quantitative computed tomography (HRpQCT) provides new insight into the impact that HOA has on bone microarchitecture and to date there have been limited in vivo studies in this area. A recent study reported on the HRpQCT changes at the second metacarpal head (MCP2) and radius between healthy individuals and HOA patients [7]. While the study was relatively small, it suggested that clinical HOA was associated with reduced trabecular and increased cortical bone mass in the MCP2 head [7]. Our study aims to extend these findings by describing the relationship between radiographic HOA and HRpQCT measures of the hand, wrist, and radius in a population-based study of older adults.

## Methods

### Participants

**Fig. 1:**
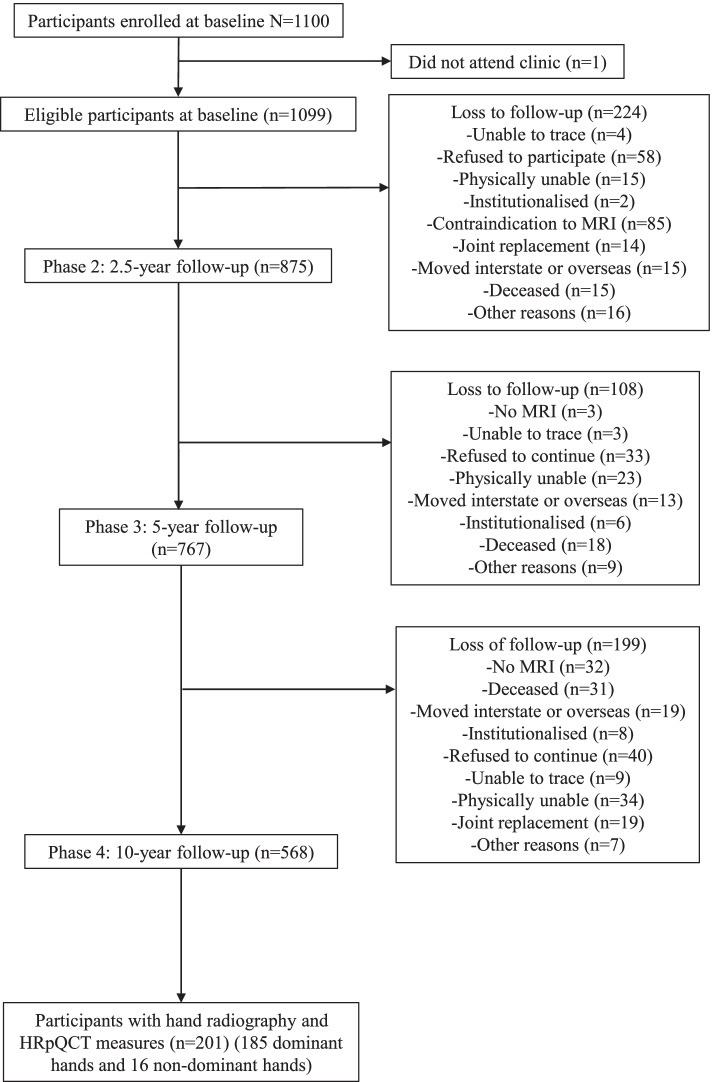
Flow-chart of study participants

The Tasmanian Older Adult Cohort (TASOAC) study is a prospective, population-based study primarily aimed at identifying factors associated with the development and progression of OA and osteoporosis in community-dwelling older adults. Participants aged 50 years and above were selected using a sex-stratified random sampling technique from the electoral roll in Southern Tasmania (population 229,000). A total of 1099 adults (response rate = 57%) consented to participate in the study. Participants were excluded if they had any implants that would prevent them from undergoing a magnetic resonance imaging (MRI) scan or they were living in a nursing home. Participants who consented to participate in the study were invited to attend a clinic at the Menzies Institute for Medical Research, Hobart, Tasmania, between March 2002 and September 2004. They were invited for follow-up clinic assessments at 2.5, 5, and 10 years after the initial clinic assessment. The 10-year follow-up included a sub-study which focused on OA of the hand. Inclusion criteria for a sub-study were that participants were agreeable to have additional radiographic and clinical hand assessment at phase 4. Exclusion criteria for the sub-study included ferromagnetic implants, claustrophobia, and inability to maintain a head first, prone, arm up (superman position) without movement for the duration of the MRI scan. Multiple imaging modalities were performed on a consecutive sample including ultrasound, radiography, MRI, and HRpQCT. Therefore, this study includes 201 participants with hand radiography and HRpQCT measurements. A flow chart of study participants is given in Fig. 1. The study was approved by the Southern Tasmanian Health and Medical Human Research Ethics Committee, and written informed consent was obtained from all participants.

### HRpQCT measures at the distal and proximal 2nd DIP, the distal and proximal 2nd PIP, the 1st CMC joint, and the distal radius

**Fig. 2:**
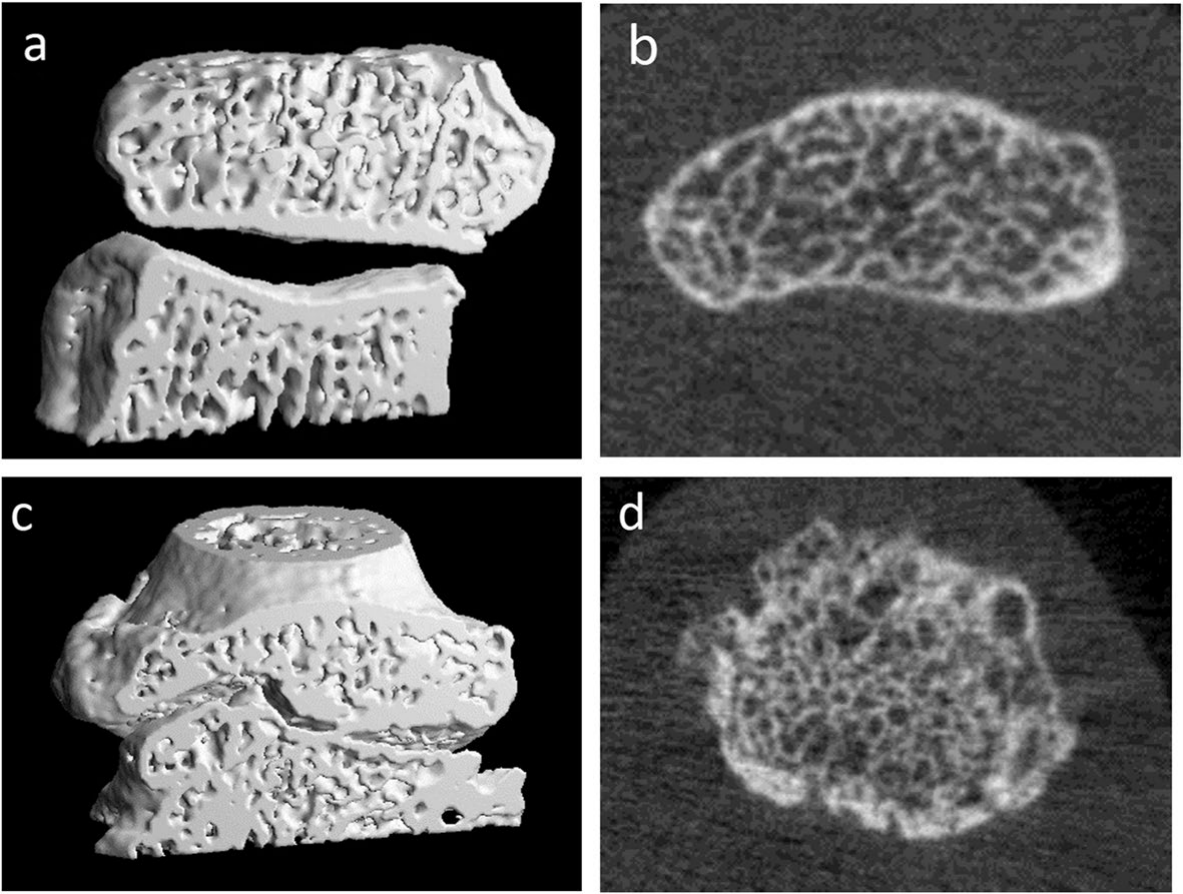
Example HRpQCT scans of the right 2nd DIP joint. **a**, **b** 3D and axial images (respectively) from a participant (male, 73 years) with an osteophyte score grade of 0. **c**, **d** 3D and axial images (respectively) from a participant (male, 76 years) with an osteophyte score of 3

Unilateral HRpQCT measurements were performed on the distal and proximal site of 2nd DIP joint (Fig. 2), the distal and proximal site of 2nd PIP joint (Supplementary Figure 1), the 1st CMC joint (Supplementary Figure 2), and the distal radius of the target hand, referred to as the assessed joint. We assessed these hand joints, the 1st CMC joint, the 2nd DIP and PIP joints, as they were in a line which made scanning technically easier and this in plane presentation was used for a parallel study of MRI. The target hand for each participant was the dominant hand unless HRpQCT was contraindicated, in which case the contralateral hand was examined, or the participant was excluded from this study (185 dominant hands and 16 non-dominant hands were scanned and assessed).

HRpQCT (XtremeCT, Scanco Medical AG, Bruttisellen, Switzerland) measurements were performed by immobilizing the target forearm with a flat position in the carbon fiber shell. A dorsal-palmar scout-view X-ray image was obtained to define the scan region of 2nd DIP, 2nd PIP, and 1st CMC [8]. The reference point was defined as the midpoint of joint. Scans were performed in a 9.02mm with 110 slices of region of interest (ROI) at the 2nd DIP, 2nd PIP, and 1st CMC using the manufacturer’s standard patient settings for image acquisition (tube potential of 60 kVp, tube current of 900 μA, 100 ms integration time, isotropic voxel size of 82μm) [8, 9]. Subchondral bone including subchondral bone plate and subchondral trabecular bone has been mostly defined as the bone components lying beneath calcified cartilage, and deeper bone structure [10, 11]. Subchondral regions were evaluated for 20 slices in both distal and proximal sites of the 2nd DIP and 2nd PIP joints, and 110 slices for the 1st CMC using the standard protocol provided by the manufacturer. Due to marked variations in joint shape, the 1st CMC was not separated by the distal and proximal site. The same HRpQCT measurements were performed on the distal radius. The ROI of 9.02 mm (110 CT slices) was at the standardized distance of 9.5 mm from the manually positioned reference line at the end plate of the distal radius.

The semi-automatic contours were created by the device automatically and checked carefully by an operator to segment subchondral plate and trabecular bone. The delineation of the cortical and trabecular compartments was defined automatically using a filter and global fixed threshold to extract the mineralized bone phase using the standard protocol of Scanco software [12, 13]. The reproducibility errors for segmentation and quantification expressed as root mean square coefficients of variation ranged from 0.54 to 3.98% and were <1.5% for volumetric bone mineral density (vBMD) [14]. All scans were graded for motion artifacts using a grading scale from one (no motion) to five (significant blurring and discontinuities). Grading 1–3 could be used. While higher grading of four or higher was rescanned on the day [15].

Bone parameters were derived from the default device using the scanner manufacturer’s software (IPL v5.08b, Scanco Medical AG). Primary bone parameters were measured using the direct model of the device, whereas derived measures were identified with superscript “d” [7, 16]. The following bone parameters were determined for the 1st CMC, the distal and proximal subchondral regions of the 2nd DIP and PIP joints (i.e., the distal and proximal 2nd DIP, the distal and proximal 2nd PIP), and the distal radius: bone areas (total bone area, cortical area, trabecular area), bone density (total, cortical and trabecular vBMD), cortical bone microarchitecture (cortical thickness, and cortical perimeter), and trabecular microarchitecture (trabecular bone volume fraction, trabecular number, trabecular thickness, trabecular separation, and inhomogeneity of trabecular network). Bone parameters and definitions were shown in Supplementary Table 1. The rationale for including all the outcome measures of interest of this study was hypothesis-generating.

### Radiographic hand assessment of the 2nd DIP, 2nd PIP, and 1st CMC joints

Radiography is considered the gold standard for the imaging of OA joints. Consequently, a digital posteroanterior radiograph of both hands was acquired. This scan provides a two-dimensional image with high spatial resolution for fine detail of the bones of the hand [17] allowing for the diagnosis of radiographic HOA. For this component of the study, the 2nd DIP joint, 2nd PIP joint, and the 1st CMC joints of the target hand were scored using the OARSI atlas [18] for the presence or absence of osteophytes (0–3) (distal and proximal separately) and joint space narrowing (JSN) (0–3) (the whole joint) by consensus by 2 readers who were a trained rheumatologist and radiographer respectively. The intra-observer agreement of hand OA assessment were published previously. Intra-observer reliability was excellent for both osteophytes and JSN scores (osteophyte score 0.98, JSN 0.94), assessed one week apart (*n* = 45) [19].

### Clinical hand assessment

Bilateral clinical examination of the hands for tenderness, soft tissue swelling, hard tissue enlargement, and deformity was performed and assessed as absent or present by a trained nurse. The assessed joints were the fifteen joints of the hand that include the 1st CMC joint, 1st to 5th metacarpophalangeal joints, 1st to 5th PIP joints, and 2nd to 5th DIP joints. Pain in the joints in the target hand was determined by questioning the participants if they had pain in each individual joint in the preceding seven days. Tenderness was assessed by the examiner exerting pressure onto the joint using their thumb and index finger sufficient to produce whitening of the examiners nail bed [20]. Soft tissue swelling was assessed visually and by palpation by the examiner ascertaining whether the joint appeared swollen. Nodules were assessed by manual examination of each joint and deformity was determined by the appearance of any deviation in the joint from the sagittal plane. Clinical HOA was diagnosed according to the American College of Rheumatology (ACR) classification criteria for hand OA [21] based upon the results of the clinical hand examination. The intra-observer reliability of each of the abnormalities (deformity, nodules, swollen, tenderness) at the joint level was assessed with at least a 1-week interval between the readings, using a kappa statistic in 10 participants with fair to substantial reliability (*κ* ranging from 0.376 to 0.688) [22].

### Anthropometry

Height was measured to the nearest 0.1 cm (with the participant having removed shoes, socks, and headwear) by stadiometer. Weight was measured to the nearest 0.1 kg (with the participant having removed shoes, socks, and bulky clothing) by calibrated electronic scales. Body mass index (BMI, kg/m^2^) was calculated by dividing weight in kilograms by height in meters squared.

### Statistical analyses

*T*-tests or chi-squared tests were used to assess the differences between continuous and categorical characteristics, as appropriate, of the participants included in this sub-study (*n* = 201) and the remaining 10-year follow-up sample (*n* = 367).

Linear-mixed effect models were first run to examine the relationships between osteophyte and JSN scores with HRpQCT measures, including data for all five ROIs in one hand (the proximal and distal 2nd PIP, the proximal and distal 2nd DIP, and the 1st CMC) using fixed effects for age, sex, and BMI, and random intercepts for ROIs. Correlations between observations on the same individual were accounted for using clustered sandwich estimator with robust standard error. In these linear-mixed effect models (both the osteophyte and JSN model), there were significant interactions between joint sites (proximal/distal) and radiographic HOA (osteophyte and JSN scores) on all HRpQCT measures, so all analyses were stratified by proximal (including the proximal 2nd DIP and proximal 2nd PIP) and distal site of joints (including the distal 2nd DIP and distal 2nd PIP), and 1st CMC.

Linear-mixed effect models including fixed effects for age, sex, and BMI, and random intercepts for ROIs were used to examine the relationships between osteophyte and JSN scores with HRpQCT measures at the distal site of joints (i.e., distal 2nd DIP and distal 2nd PIP). Similarly, linear-mixed effect models including fixed effects for age, sex, and BMI, and random intercepts for ROIs were used to examine the relationships between osteophyte and JSN scores with HRpQCT measures at the proximal site of joints (i.e., proximal 2nd DIP and proximal 2nd PIP).

A separate multivariable linear regression model was run for the 1st CMC joint which adjusted for age, sex, and BMI. Lastly, a further linear regression model was run to examine the relationship between osteophyte and JSN scores in the hand (1st CMC, 2nd DIP, 2nd PIP) with HRpQCT measures at the distal radius adjusted for age, sex and BMI.

Additional analyses for distal DIP, proximal DIP, distal PIP, and proximal PIP were run using separate multivariable linear regression models and adjustment for age, sex, and BMI.

All HRpQCT measures were standardized. All statistical analyses were performed using StataSE 16.1 for Windows (StataCorp, College Station, TX, USA). A two-side *p*-value < 0.05 was considered statistically significant. The family-wise error rate was used to control the overall false-positive rate for multiple testing [23]. In this study, the adjusted alpha level was 0.0167. Using the adjusted alpha level, most results remained statistically significant. Thus, we presented all data.

## Results

The subgroup characteristics of the 201 TASOAC participants that underwent HRpQCT assessment are presented in Tables 1 and 2. The average age was 72 with 46% being female. Despite being a community-based sample, there was a high prevalence of clinical HOA (64%). Radiographic hand OA at 1st CMC, 2nd DIP, and 2nd PIP was present in 66% of participants. Seventy-seven percent had osteophytes, and 29% had JSN at 1st CMC, 2nd DIP, and 2nd PIP. There were no significant differences between those included in this study (*n* = 201) and the remaining 10-year TASOAC (*n* = 367) sample for age, BMI, sex, ACR clinical HOA, or radiographic HOA (data not shown).

**Table 1:** Characteristics of participants included in this TASOAC sub-study

	*N* = 201^*^
Age (years)	72.19 ± 6.52
Sex (% female)	46%
Weight (kg)	77.52 ± 14.38
BMI (kg/m^2^)	27.75 ± 4.27
Self-reported osteoporosis (%)	6%
ACR hand osteoarthritis present	64%
Osteophytes present at 1st CMC, 2nd DIP, and 2nd PIP	77%
Average osteophytes score of 1st CMC, 2nd DIP, and 2nd PIP (1–3)	1.62 ± 0.79
Joint Space Narrowing present at 1st CMC, 2nd DIP, and 2nd PIP	29%
Average joint space narrowing score of 1st CMC, 2nd DIP, or 2nd PIP (1–3)	2.05 ± 0.79
Clinical hand assessment of 1st CMC, 2nd DIP, and 2nd PIP	
Swollen present	2%
Tender present	18%
Nodules present	79%
Deformity present	31%

*CMC* carpometacarpal joint, *DIP* distal interphalangeal joint, *PIP* proximal interphalangeal joint

^*^Values are mean ± SD except for percentages

**Table 2:** HRpQCT indices for each hand joint ROI (*N* = 201)

	1st CMC	Distal 2nd DIP	Proximal 2nd DIP	Distal 2nd PIP	Proximal 2nd PIP
**Areas and density**					
Total bone area (mm^2^)	278.03 ± 80.02	55.11 ± 12.74	54.32 ± 12.59	93.07 ± 15.24	70.89 ± 15.60
Cortical area (mm^2^)	35.16 ± 19.24	13.09 ± 6.70	18.45 ± 7.98	18.98 ± 11.93	18.43 ± 10.07
Trabecular area (mm^2^)	186.21 ± 50.41	33.71 ± 10.50	28.15 ± 13.14	63.95 ± 15.04	42.51 ± 14.05
Total volumetric bone density (mg HA/cm^3^)	312.77 ± 46.00	439.57 ± 73.54	510.31 ± 87.06	415.58 ± 74.63	469.84 ± 73.64
Cortical volumetric bone density (mg HA/cm^3^)	474.04 ± 60.08	635.53 ± 70.48	655.23 ± 70.33	616.47 ± 73.56	615.45 ± 68.28
Trabecular volumetric bone density (mg HA/cm^3^)	236.41 ± 34.39	309.66 ± 46.07	360.88 ± 43.86	318.63 ± 40.17	369.82 ± 45.17
**Cortical bone microarchitecture**					
Cortical thickness (mm)	0.36 ± 0.16	0.41 ± 0.20	0.55 ± 0.25	0.47 ± 0.28	0.46 ± 0.24
Cortical perimeter (mm)	94.43 ± 17.53	31.84 ± 3.66	34.36 ± 5.46	40.28 ± 3.52	39.62 ± 6.05
**Trabecular microarchitecture**					
Trabecular bone volume fraction^a^ (%)	0.20 ± 0.03	0.26 ± 0.04	0.30 ± 0.04	0.27 ± 0.03	0.31 ± 0.04
Trabecular number (1/mm)	2.33 ± 0.33	2.35 ± 0.29	2.27 ± 0.33	2.47 ± 0.21	2.45 ± 0.25
Trabecular thickness (mm)	0.09 ± 0.01	0.11 ± 0.02	0.13 ± 0.02	0.11 ± 0.01	0.13 ± 0.02
Trabecular separation (mm)	0.36 ± 0.08	0.32 ± 0.07	0.32 ± 0.07	0.30 ± 0.04	0.29 ± 0.04
Inhomogeneity of trabecular network^a^ (mm)	0.24 ± 0.17	0.15 ± 0.09	0.17 ± 0.10	0.13 ± 0.05	0.13 ± 0.06

Values are mean ± SD

*CMC* carpometacarpal joint, *DIP* distal interphalangeal joint, *PIP* proximal interphalangeal joint

^a^Parameters were calculated using the derived measurement method

Table 3 shows the standardized beta-coefficients between site-specific osteophyte/JSN scores and HRpQCT measures at 1st CMC joint, distal, and proximal sites. Osteophyte scores at the proximal site were more strongly associated with HRpQCT measures compared to the distal site with positive associations for indices of bone size (total, cortical, and trabecular bone area, and cortical perimeter) and negative associations for total, cortical, and trabecular vBMD. There was a decrease in trabecular number and bone volume fraction with increasing osteophyte scores as well as an increase in trabecular separation and inhomogeneity. At 1st CMC joint, osteophyte scores were positively associated with indices of bone size, trabecular thickness, and trabecular inhomogeneity.

**Table 3:** Standardized beta-coefficients for the associations of site-specific osteophyte and joint space narrowing scores with HRpQCT measures (per SD) stratified by 1st CMC, distal, and proximal sites (*N* = 201)

	Osteophyte scores			Joint space narrowing scores		
	1st CMC *β* (95% CI)^a^	Distal site *β* (95% CI)^b^	Proximal site *β* (95% CI)^b^	1st CMC *β* (95% CI)^a^	Distal site *β* (95% CI)^b^	Proximal site *β* (95% CI)^b^
**Areas and density**						
Total bone area	**0.35 (0.26, 0.44)**	**0.07 (0.05, 0.10)**	**0.21 (0.17, 0.24)**	**0.38 (0.28, 0.48)**	**0.03 (0.01, 0.04)**	**0.09 (0.07, 0.10)**
Cortical area	**0.54 (0.38, 0.71)**	**0.14 (0.01, 0.28)**	**0.16 (0.01, 0.31)**	**0.65 (0.48, 0.83)**	0.06 (−0.01, 0.14)	0.03 (−0.03, 0.10)
Trabecular area	**0.35 (0.26, 0.45)**	**0.06 (0.02, 0.10)**	**0.20 (0.15, 0.26)**	**0.36 (0.26, 0.47)**	0.01 (−0.01, 0.04)	**0.09 (0.06, 0.11)**
Total vBMD	0.06 (−0.01, 0.13)	−0.03 (−0.19, 0.12)	**−0.48 (−0.69, −0.27)**	**0.08 (0.01, 0.16)**	−0.03 (−0.12, 0.06)	**−0.26 (−0.35, −0.17)**
Cortical vBMD	0.07 (−0.02, 0.17)	−0.04 (−0.20, 0.12)	**−0.49 (−0.69, −0.30)**	0.07 (−0.04, 0.17)	−0.06 (−0.15, 0.03)	**−0.24 (−0.33, −0.16)**
Trabecular vBMD	0.06 (−0.02, 0.14)	−0.10 (−0.24, 0.03)	**−0.53 (−0.70, −0.36)**	**0.09 (0.01, 0.18)**	**−0.09 (−0.17, −0.01)**	**−0.28 (−0.35, −0.21)**
**Cortical bone microarchitecture**						
Cortical thickness	**0.23 (0.14, 0.32)**	0.09 (−0.11, 0.30)	−0.17 (−0.43, 0.09)	**0.29 (0.19, 0.38)**	0.02 (−0.10, 0.13)	−**0.16 (**−**0.27,** −**0.04)**
Cortical perimeter	**0.26 (0.19, 0.34)**	**0.09 (0.06, 0.12)**	**0.31 (0.26, 0.37)**	**0.26 (0.17, 0.35)**	**0.05 (0.03, 0.06)**	**0.15 (0.13, 0.17)**
**Trabecular microarchitecture**						
Tb.BV/TV^c^	0.07 (−0.01, 0.15)	−0.10 (−0.24, 0.03)	**−0.53 (−0.70, −0.36)**	**0.10 (0.01, 0.18)**	**−0.09 (−0.17, −0.01)**	**−0.28 (−0.35, −0.21)**
Trabecular number	−0.03 (−0.19, 0.12)	**−0.27 (−0.45, −0.09)**	**−0.58 (−0.85, −0.31)**	0.01 (−0.16, 0.18)	**−0.27 (−0.36, −0.16)**	**−0.34 (−0.46, −0.22)**
Trabecular thickness	**0.10 (0.05, 0.16)**	0.11 (−0.03, 0.25)	−0.04 (−0.25, 0.16)	**0.11 (0.05, 0.17)**	**0.11 (0.03, 0.19)**	−0.03 (−0.12, 0.06)
Trabecular separation	0.10 (−0.09, 0.26)	**0.23 (0.06, 0.41)**	**0.64 (0.41, 0.87)**	0.05 (−0.14, 0.24)	**0.27 (0.17, 0.37)**	**0.33 (0.23, 0.43)**
Tb.1/N.SD^c^	**0.23 (0.01, 0.45)**	**0.29 (0.15, 0.43)**	**0.46 (0.27, 0.66)**	0.17 (−0.08, 0.41)	**0.21 (0.13, 0.29)**	**0.22 (0.13, 0.30)**

Beta coefficients represent a 1 unit increase in osteophyte/JSN score per SD change in HRpQCT measure. Bold denotes statistical significance. Distal site: distal 2nd distal interphalangeal joint, distal 2nd proximal interphalangeal joint. Proximal site: proximal 2nd distal interphalangeal joint, and proximal 2nd proximal interphalangeal joint

*SD* standard deviation, *CI* confidence interval, *CMC* carpometacarpal joint, *vBMD* volumetric bone density, *Tb.BV/TV* trabecular bone volume fraction, *Tb.1/N.SD* inhomogeneity of trabecular network

^a^Multivariable linear regression adjusting for age, sex, and BMI

^b^Mixed-effects model including fixed effects for age, sex, BMI, and random intercepts for ROIs

^c^Parameters were calculated using the derived measurement method

Similar to osteophytes, JSN scores at the proximal site were more strongly associated with HRpQCT measures compared to the distal site (Table 3). There were positive associations for indices of bone size (total, trabecular bone area, and cortical perimeter, but inconsistent for cortical area) and negative associations for total, cortical, and trabecular vBMD. There was a decrease in trabecular number and bone volume fraction with increasing JSN scores as well as an increase in trabecular separation and inhomogeneity. At 1st CMC joint, JSN scores were positively associated with indices of bone size, vBMD measures (total and trabecular vBMD), trabecular bone volume fraction, and thickness. Additional analyses, running separate models for distal DIP, proximal DIP, distal PIP, and proximal PIP, show similar findings to the linear-mixed models, indicating the associations are stronger at the proximal site (Supplementary Table 2 and 3).

The associations between osteophyte/JSN and HRpQCT measures remained statistically significant after further adjustment for current smoking status, alcohol consumption, physical activity, and occupational impact on hand joints (Supplementary Table 4). We performed sensitivity analyses with further adjustment for self-reported diagnosis of osteoporosis and found similar results (Supplementary Table 5).

There were no significant associations between osteophyte and JSN scores in the hand with HRpQCT measures at the distal radius (Supplementary Table 6).

## Discussion

This study addresses an important evidence gap by describing the relationship between radiographic HOA, a commonly used modality in clinical practice, and HRpQCT measures. As osteophyte and JSN severity increases, there are increases in bone size indices (total and trabecular bone area), cortical perimeter, and trabecular separation with the trabecular network becoming less homogeneous. In contrast, local bone density (vBMD) decreases. Furthermore, there are less trabeculae per mm and the trabecular bone volume decreases. These changes appear to vary by joint site, as shown by the novel finding that the associations were stronger for the proximal site (proximal 2nd DIP and proximal 2nd PIP) compared to the distal site (distal 2nd DIP and distal 2nd PIP). Overall, these findings indicate substantial changes in the finger subchondral trabecular bone in radiographic HOA. Further research would be warranted to validate and extend our findings.

It has long been believed that OA pathogenesis has an inverse relationship with age-related bone loss [1]. However, HOA subchondral bone changes have not previously been described in vivo using HRpQCT, apart from a small recent case-control study which considered clinical HOA [7]. In our study, the expansion in subchondral trabeculae with osteophyte formation supports the hypothesis that increased area of subchondral trabecular bone plays an important role in the pathogenesis of radiographic OA [3]. In addition, local bone vBMD decreased with osteophyte formation and JSN. Similar results have been observed in other studies on HOA, which used micro-CT in animal post mortem models or unenhanced CT algorithms/HRpQCT in vivo [7, 24–27]. Thus, the current HRpQCT results further support that the association with vBMD is negative rather than positive in radiographic HOA [28, 29]. However, these results are contrary to previous dual-energy X-ray absorptiometry (DXA) results which show increased areal density in the hip and spine OA [30, 31], and positive relationships between subchondral bone density and knee OA structural measures, such as osteophytes, JSN, and cartilage defects [32, 33]. This can be explained by the limitations of two dimensional DXA, which may be confounded by bone area change, as has been reported at the spine [31]. Another possible explanation is different loading forces between knee and hand joints. Therefore, the current study suggests that the major subchondral trabecular changes in radiographic HOA are for both bone size and vBMD, which appear to be in the opposite directions.

Our results contrast somewhat with a recent study which reported HRpQCT changes between healthy individuals and HOA patients [7]. This study reported reduced trabecular vBMD and increased cortical vBMD in hand OA patients fulfilling the ACR criteria for clinical HOA [7]. Interestingly in subgroup analysis with radiographic features (e.g., erosions and osteophytes), no associations were found with trabecular or cortical bone density. These differences could be attributed to the definition used for HOA (clinical versus radiographic). The author also reported a sex interaction, which we did not observe in our current study.

Our study has also shown that there were subchondral trabecular microarchitecture changes adjacent to the 2nd PIP and 2nd DIP joints with osteophyte formation. We observed a decreased number of trabeculae, decreased bone volume fraction, and increased separation and inhomogeneity, which are consistent with previous studies in knee OA using MRI in vivo and micro-CT/HRpQCT in animal models [34–37], and recent studies in anterior cruciate ligament reconstruction knees using HRpQCT in vivo [38, 39]. However, opposite evidence on the characteristics of subchondral trabecular changes with progression of OA has also been reported in knee OA [2, 40]. This inconsistency may be explained by knee specimens not being studied in vivo (collected during total knee arthroplasty surgery) which is unlikely to reflect “early” stage knee OA. Another possible explanation is higher loading in weight-bearing joints lead to compensatory thicker and denser subchondral trabecular bone than that in non-weight-bearing joints [41, 42]. Overall, our findings suggest that alterations occur in subchondral trabecular bone. Patients with radiographic hand OA may have higher levels of bone resorption and perforation of trabeculae or it may be that people with poor trabecular bone are at a higher risk of hand OA. Further longitudinal HRpQCT study may be required to investigate the mechanism.

A unique finding in this study is that the associations seen between osteophyte and JSN scores with HRpQCT measures were consistently stronger for the proximal site compared to the distal site. A possible explanation is some features associated with progressive disease such as bone edema and osteophyte development have been mostly observed on the proximal PIP site compared to the DIP site in early hand OA [43]. However, it seems that findings do vary depending on the population. For example, a cross-sectional study including 11 women with long-standing erosive HOA reported that MRI-defined bone marrow lesions correlated with the development of erosive HOA, and some associations were stronger at the proximal site [44]. In contrast, a study in 21 women without radiographic HOA reported that trabecular texture in the distal region was associated with MRI-defined osteophytes, but no associations were found for the proximal site [45]. To date, studies have been small and further research is needed to confirm these results in populations both with and without HOA.

Furthermore, it is interesting to note that, in this study, associations of osteophyte severity and vBMD were positive for some 1st CMC indices, while these were consistently and strongly negative in other assessed regions. The discrepancy may be explained by the difference of local mechanical loading history and use patterns in different regions of hand joints [24, 46]. In line with a previous study on cadaveric thumb CMC OA [47], significant changes were observed at trabecular bone thickness, Tb.TV/BV, and inhomogeneity of trabecular network with osteophyte formation and JSN at 1st CMC. Irrespective of the direction of the association, these findings suggest that subchondral trabecular bone is a significant component of the pathogenesis of HOA.

There is a hypothesis that OA is a condition that is due primarily to a systemic predisposition to mechanical stress [48], although this study did not observe associations between HOA osteophyte and JSN and radial bone microarchitecture. HOA should not rule out a possible systemic effect of OA, as this study lacks data on radial subchondral bone, genetic predisposition to OA, and forces experienced by the assessed joints.

There are some potential limitations in this study. Firstly, the cross-sectional study design cannot shed light on causal pathways. While long-term longitudinal data would be preferable, it will be some time before such studies can be completed given that HRpQCT is a recent development. Secondly, the sample size was relatively small. While this was a subsample of a larger cohort, there were no differences between those included and those not included, suggesting broader generalizability. Thirdly, it is possible that some osteophytes were overlooked due to low sensitivity of X-ray. While it would have been more sensitive to assess osteophytes on HRpQCT, this study aimed to assess the association between radiographic OA (a commonly used modality in clinical practice) and HRpQCT measures. Further research could examine the relationship between HRpQCT-detected osteophytes and microarchitecture. Fourthly, the first CMC joint may not have been fully captured and could not be separated by the proximal and distal site, due to marked variations in joint shape, which may bias estimations and limit further interpretation. Additionally, the reliability of the clinical assessment is poor. Fifthly, in this study, we used an unvalidated modification of the OARSI atlas by performing separate osteophyte scoring for the proximal and distal joint halves. We did this in order to perform a site-specific analysis with the HRpQCT measures. Further validation of the modified OARSI atlas will be required. Lastly, although the subchondral bone plate was included and evaluated in scans, the segmentation of subchondral cortical bone using the standard protocol of Scanco software may not accurately analyze the subchondral cortical bone. Further study using adaptive local thresholding technique may enhance the accuracy of HRpQCT for subchondral cortical bone [49].

In conclusion, this hypothesis generating data suggests that bone size and trabecular disorganization increase with both osteophyte formation and JSN (proximal more than distal), while local bone density (vBMD) decreases. This process appears to be primarily at the site of pathology rather than nearby unaffected bone.

## Supplementary Information

**Additional file 1: Supplementary Figure 1.** Example HRpQCT scans of right 2^nd^ PIP joint. a) and b) are 3D and axial images (respectively) from a patient (male, 78 years) with an osteophyte score grade of 0. **Supplementary Figure 2.** Example HRpQCT scans of right 1^st^ CMC joint. a) and b) are 3D and axial images (respectively) from a patient (male, 78 years) with an osteophyte score grade of 0.

**Additional file 2: Supplementary Table 1.** Bone parameters, abbreviation, unit, description and measure methods.

**Additional file 3: Supplementary Table 2.** Standardized beta-coefficients for the associations of osteophyte scores with site-specific HRpQCT measures (per SD) stratified by 1st CMC, distal and proximal DIP and PIP sites (N=201).

**Additional file 4: Supplementary Table 3.** Standardized beta-coefficients for the associations of joint space narrowing scores with site-specific HRpQCT measures (per SD) stratified by 1st CMC, distal and proximal DIP and PIP sites (N=201).

**Additional file 5: Supplementary Table 4.** Standardized beta-coefficients for the associations of site-specific osteophyte and joint space narrowing scores with HRpQCT measures (per SD) stratified by 1st CMC, distal and proximal sites (N=201) further adjustment for alcohol intake, current smoking, physical activity, occupational impact.

**Additional file 6: Supplementary Table 5.** Standardized beta-coefficients for the associations of site-specific osteophyte and joint space narrowing scores with HRpQCT measures (per SD) stratified by 1st CMC, distal and proximal sites (N=201) further adjusted for self-reported diagnosis of osteoporosis.

**Additional file 7: Supplementary Table 6.** Standardized beta-coefficients for the associations of osteophyte and joint space narrowing scores at hand joints with HRpQCT measures (per SD) at distal radius (N=201).

## Data Availability

The datasets generated and/or analyzed during the current study are available from the corresponding author on reasonable request.

## Abbreviations

OA: Osteoarthritis
HOA: Hand osteoarthritis
HRpQCT: High-resolution peripheral quantitative computed tomography
MCP2: The second metacarpal head
vBMD: Volumetric bone mineral density
TASOAC: The Tasmanian Older Adult Cohort
MRI: Magnetic resonance imaging
DIP: Distal interphalangeal joint
PIP: Proximal interphalangeal joint
CMC: Carpometacarpal joint
ROI: Region of interest
JSN: Joint space narrowing
ACR: American College of Rheumatology
CT: Computed tomography
DXA: Dual-energy X-ray absorptiometry
Tb.TV/BV: Trabecular bone volume fraction
