# Supplementary material for: Association between radiographic hand osteoarthritis and bone microarchitecture in a population-based sample
Source: Arthritis Res Ther. 2022 Sep 17;24:223. doi: 10.1186/s13075-022-02907-6 (PMC9482179; doi:10.1186/s13075-022-02907-6)
Supplement: Supplementary file 1 — Additional file 1: Supplementary Figure 1. Example HRpQCT scans of right 2nd PIP joint. a) and b) are 3D and axial images (respectively) from a patient (male, 78 years) with an osteophyte score grade of 0. Supplementary Figure 2. Example HRpQCT scans of right 1st CMC joint. a) and b) are 3D and axial images (respectively) from a patient (male, 78 years) with an osteophyte score grade of 0. [file 13075_2022_2907_MOESM1_ESM.doc]

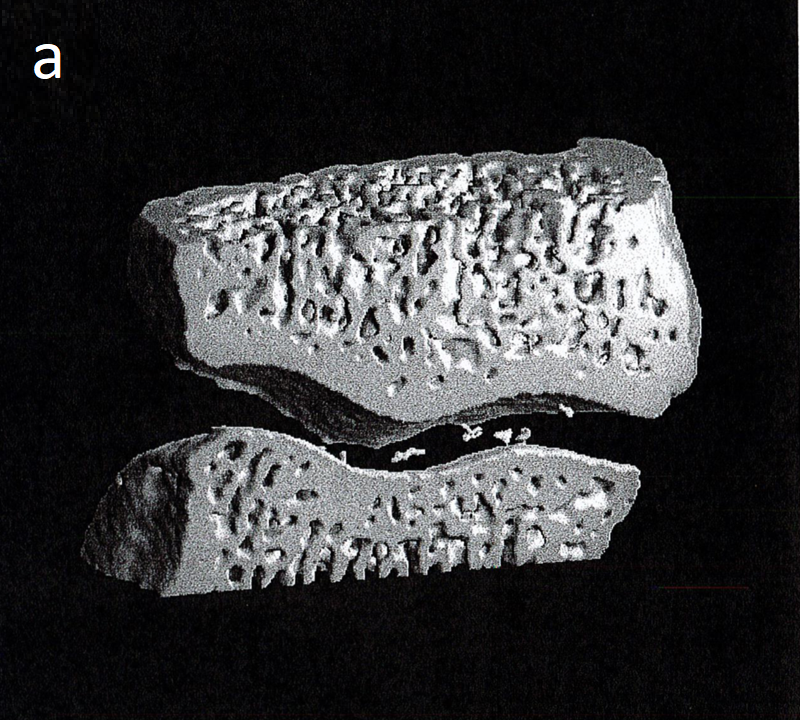

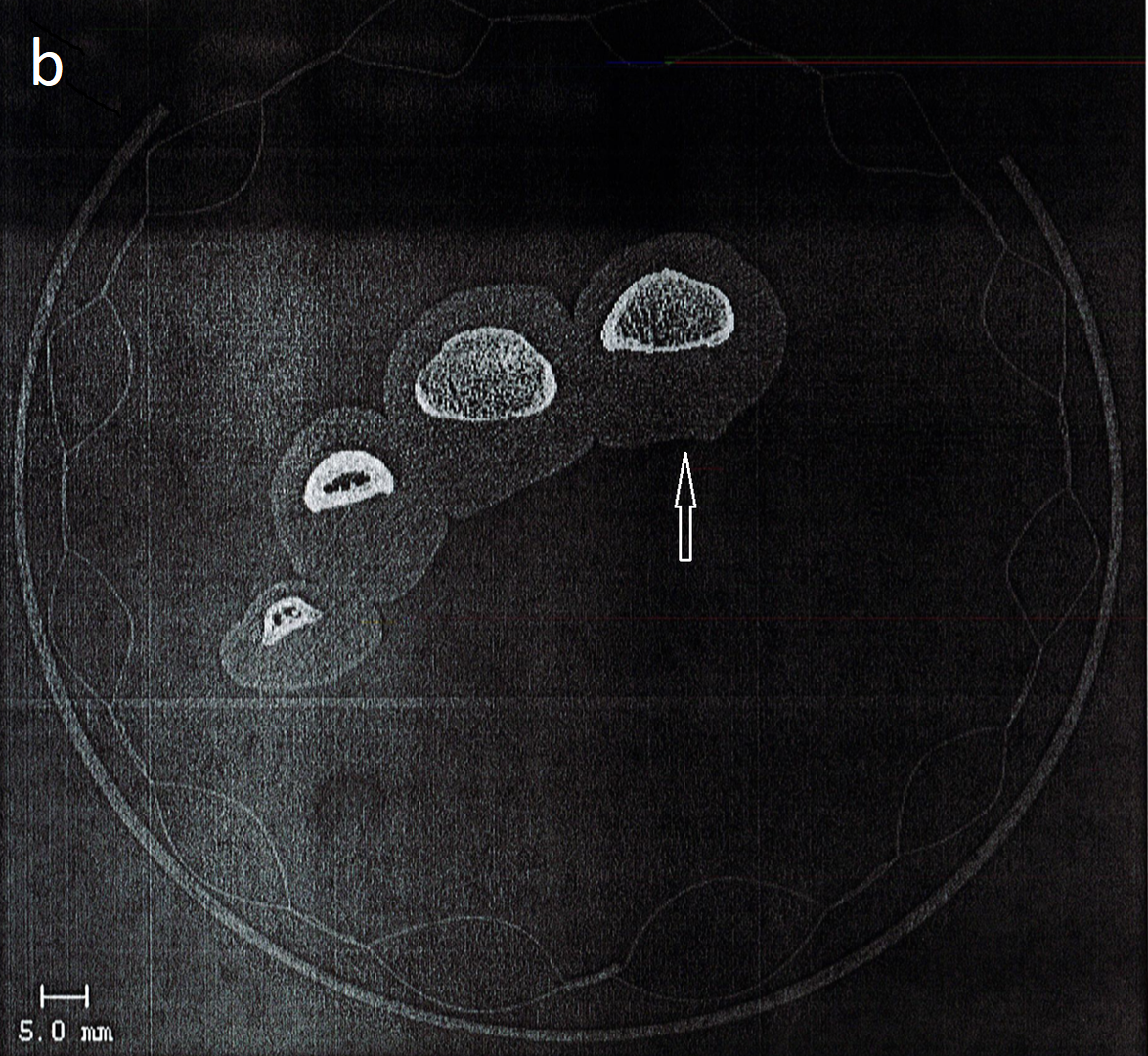


**Supplementary Figure 1.** Example HRpQCT scans of right 2^nd^ PIP joint. a) and b) are 3D and axial images (respectively) from a patient (male, 78 years) with an osteophyte score grade of 0.


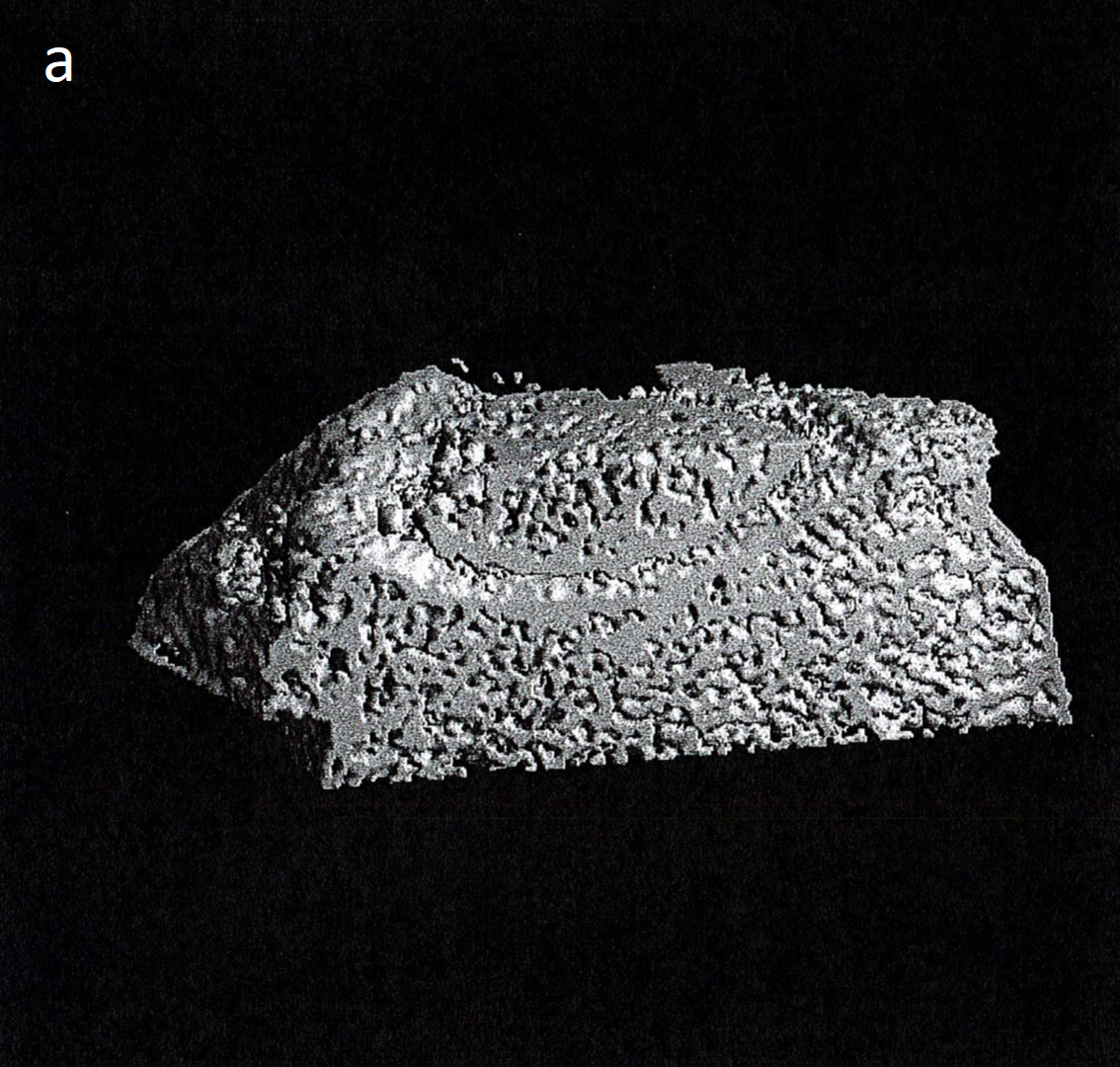

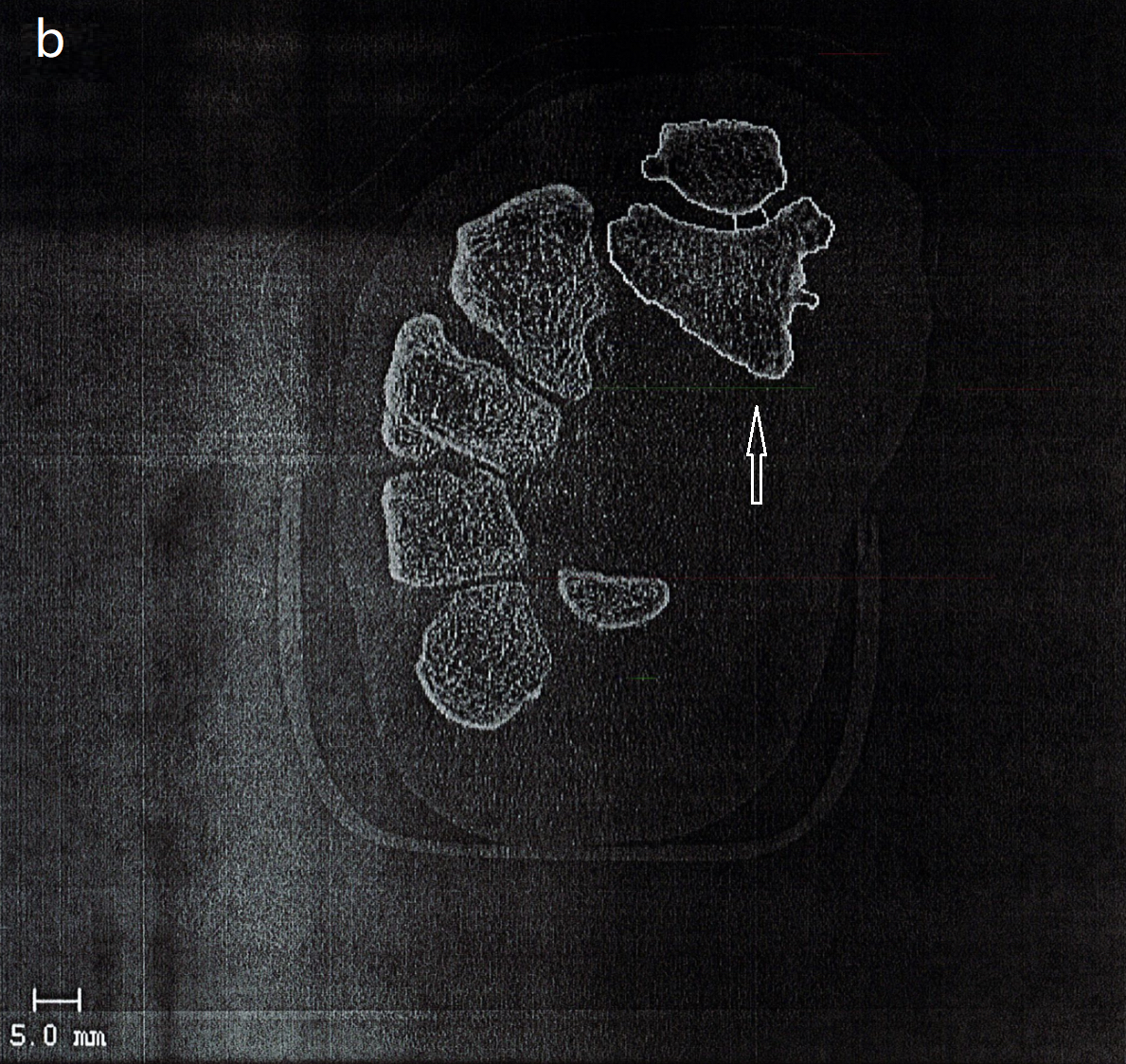


**Supplementary Figure 2.** Example HRpQCT scans of right 1^st^ CMC joint. a) and b) are 3D and axial images (respectively) from a patient (male, 78 years) with an osteophyte score grade of 0.
