# Supplementary material for: Association between radiographic hand osteoarthritis and bone microarchitecture in a population-based sample
Source: Arthritis Res Ther. 2022 Sep 17;24:223. doi: 10.1186/s13075-022-02907-6 (PMC9482179; doi:10.1186/s13075-022-02907-6)
Supplement: Supplementary file 2 — Additional file 2: Supplementary Table 1. Bone parameters, abbreviation, unit, description and measure methods. [file 13075_2022_2907_MOESM2_ESM.docx]

**Supplementary Table 1.** Bone parameters, abbreviation, unit, description and measure methods

| Parameter | Abbreviation and standard unit | Description | Methods |
| --- | --- | --- | --- |
| **Bone areas** |  |  |  |
| Total bone area | Tt.Ar (mm^2^) | The average of total cross-sectional area of selected or 110 slices | Direct model |
| Cortical area | Ct.Ar (mm^2^) | The average of total sectional area of cortical compartments | Direct model |
| Trabecular area | Tb.Ar (mm^2^) | The average of total sectional area of medullary compartments | Direct model |
| **Bone density** |  |  |  |
| Total volumetric bone density | Tt.vBMD (mg HA/cm^3^) | The average volumetric density of total bone | Direct model |
| Cortical volumetric bone density | Ct.vBMD (mg HA/cm^3^) | Average volumetric density within the  cortical compartment | Direct model |
| Trabecular volumetric bone density | Tb.vBMD (mg HA/cm^3^) | Average mineral density within the  trabecular compartment | Direct model |
| **Cortical bone microarchitecture** |  |  |  |
| Cortical thickness | Ct.Th (mm) | Average thickness of the cortical compartment | Direct model |
| Cortical perimeter | Ct.Pm (mm) | The average perimeter of selected or 110 slices | Direct model |
| **Trabecular bone microarchitecture** |  |  |  |
| Trabecular bone volume fraction | Tb.BV/TV^d^ (%) | Ratio of segmented bone volume to total  volume of the trabecular  compartment | Tb.vBMD/1200 mg HA/cm^3^)*100 |
| Trabecular number | Tb.N (1/mm) | Number of trabecular in 1 mm | Direct model |
| Trabecular thickness | Tb.Th (mm) | Average thickness of trabeculae | Direct model |
| Trabecular separation | Tb.Sp (mm) | The average separation between the trabeculae | Direct model |
| Inhomogeneity of trabecular network | Tb.1/N.SD^d^ (mm) | Measure of inhomogeneity of trabecular  separation | Standard Deviation of 1/Tb.N |

^d^ parameters were calculated using the derived measurement method.
