# Supplementary material for: Association between radiographic hand osteoarthritis and bone microarchitecture in a population-based sample
Source: Arthritis Res Ther. 2022 Sep 17;24:223. doi: 10.1186/s13075-022-02907-6 (PMC9482179; doi:10.1186/s13075-022-02907-6)
Supplement: Supplementary file 3 — Additional file 3: Supplementary Table 2. Standardized beta-coefficients for the associations of osteophyte scores with site-specific HRpQCT measures (per SD) stratified by 1st CMC, distal and proximal DIP and PIP sites (N=201). [file 13075_2022_2907_MOESM3_ESM.docx]

**Supplementary Table 2:** Standardized beta-coefficients for the associations of osteophyte scores with site-specific HRpQCT measures (per SD) stratified by 1st CMC, distal and proximal DIP and PIP sites (N=201)

|  | 1^st^ CMC  β (95% CI)* | Distal 2^nd^ DIP  β (95% CI)* | Proximal 2^nd^ DIP  β (95% CI)* | Distal 2^nd^ PIP  β (95% CI)* | Proximal 2^nd^ PIP  β (95% CI)* |
| --- | --- | --- | --- | --- | --- |
| **Areas and density** |  |  |  |  |  |
| Total bone area | **0.35 (0.26, 0.44)** | **0.07 (0.03, 0.10)** | **0.16 (0.12, 0.20)** | **0.08 (0.04, 0.12)** | **0.31 (0.25, 0.38)** |
| Cortical area | **0.54 (0.38, 0.71)** | 0.16 (0.03, 0.29) | -0.03 (-0.19, 0.13) | 0.11 (-0.13, 0.36) | **0.70 (0.37, 1.02)** |
| Trabecular area | **0.35 (0.26, 0.45)** | **0.05 (0.002, 0.09)** | **0.20 (0.13, 0.26)** | 0.07 (-0.0009, 0.14) | **0.22 (0.11, 0.33)** |
| Total vBMD | 0.06 (-0.01, 0.13) | 0.01 (-0.20, 0.23) | **-0.61 (-0.86, -0.35)** | -0.11 (-0.35, 0.12) | -0.13 (-0.51, 0.25) |
| Cortical vBMD | 0.07 (-0.02, 0.17) | -0.02 (-0.23, 0.20) | **-0.63 (-0.84, -0.41)** | -0.09 (-0.33, 0.15) | -0.06 (-0.45, 0.33) |
| Trabecular vBMD | 0.06 (-0.02, 0.14) | -0.02 (-0.22, 0.19) | **-0.57 (-0.76, -0.37)** | -0.18 (-0.37, 0.01) | **-0.43 (-0.77, -0.08)** |
| **Cortical bone microarchitecture** |  |  |  |  |  |
| Cortical thickness | **0.23 (0.14, 0.32)** | 0.16 (-0.08, 0.39) | **-0.39 (-0.69, -0.09)** | 0.03 (-0.32, 0.38) | 0.45 (-0.05, 0.94) |
| Cortical perimeter | **0.26 (0.19, 0.34)** | **0.09 (0.05, 0.13)** | **0.27 (0.21, 0.33)** | **0.08 (0.05, 0.12)** | **0.41 (0.31, 0.52)** |
| **Trabecular microarchitecture** |  |  |  |  |  |
| Tb.BV/TV^d^ | 0.07 (-0.01, 0.15) | -0.02 (-0.22, 0.19) | **-0.57 (-0.76, -0.37)** | -0.18 (-0.37, 0.01) | **-0.43 (-0.77, -0.08)** |
| Trabecular number | -0.03 (-0.19, 0.12) | **-0.32 (-0.61, -0.04)** | **-0.55 (-0.91, -0.20)** | -0.19 (-0.42, 0.04) | **-0.56 (-1.01, -0.12)** |
| Trabecular thickness | **0.10 (0.05, 0.16)** | 0.20 (-0.02, 0.43) | -0.09 (-0.36, 0.18) | -0.04 (-0.22, 0.14) | -0.04 (-0.38, 0.30) |
| Trabecular separation | 0.10 (-0.09, 0.26) | 0.26 (-0.04, 0.57) | **0.66 (0.33, 0.99)** | **0.18 (0.01, 0.35)** | **0.51 (0.19, 0.84)** |
| Tb.1/N.SD^d^ | **0.23 (0.01, 0.45)** | **0.32 (0.07, 0.57)** | **0.43 (0.15, 0.71)** | **0.22 (0.09, 0.35)** | **0.47 (0.20, 0.73)** |

Beta coefficients represent a 1 unit increase in osteophyte score per SD change in HRpQCT measure.

*Multivariable linear regression adjusting for age, sex, and BMI.

^d^ parameters were calculated using the derived measurement method.

Bold denotes statistical significance.

Abbreviations: SD: standard deviation; CI: confidence interval; CMC: carpometacarpal joint; DIP: distal interphalangeal joint; PIP: proximal interphalangeal joint; vBMD: volumetric bone density, Tb.BV/TV: Trabecular bone volume fraction, Tb.1/N.SD: Inhomogeneity of trabecular network.
