# Supplementary material for: Association between radiographic hand osteoarthritis and bone microarchitecture in a population-based sample
Source: Arthritis Res Ther. 2022 Sep 17;24:223. doi: 10.1186/s13075-022-02907-6 (PMC9482179; doi:10.1186/s13075-022-02907-6)
Supplement: Supplementary file 4 — Additional file 4: Supplementary Table 3. Standardized beta-coefficients for the associations of joint space narrowing scores with site-specific HRpQCT measures (per SD) stratified by 1st CMC, distal and proximal DIP and PIP sites (N=201). [file 13075_2022_2907_MOESM4_ESM.docx]

**Supplementary Table 3:** Standardized beta-coefficients for the associations of joint space narrowing scores with site-specific HRpQCT measures (per SD) stratified by 1st CMC, distal and proximal DIP and PIP sites (N=201)

|  | 1^st^ CMC  β (95% CI)* | Distal 2^nd^ DIP  β (95% CI)* | Proximal 2^nd^ DIP  β (95% CI)* | Distal 2^nd^ PIP  β (95% CI)* | Proximal 2^nd^ PIP  β (95% CI)* |
| --- | --- | --- | --- | --- | --- |
| **Areas and density** |  |  |  |  |  |
| Total bone area | **0.38 (0.28, 0.48)** | **0.02 (0.004, 0.04)** | **0.06 (0.05, 0.08)** | **0.04 (0.00, 0.07)** | **0.18 (0.15, 0.22)** |
| Cortical area | **0.65 (0.48, 0.83)** | 0.01 (-0.05, 0.07) | -0.04 (-0.11, 0.03) | **0.25 (0.04, 0.46)** | **0.36 (0.18, 0.53)** |
| Trabecular area | **0.36 (0.26, 0.47)** | 0.02 (-0.002, 0.04) | **0.08 (0.06, 0.11)** | -0.02 (-0.09, 0.04) | **0.12 (0.06, 0.18)** |
| Total vBMD | **0.08 (0.01, 0.16)** | -0.07 (-0.17, 0.03) | **-0.29 (-0.40, -0.18)** | 0.07 (-0.13, 0.28) | -0.14 (-0.34, 0.06) |
| Cortical vBMD | 0.07 (-0.04, 0.17) | -0.10 (-0.20, 0.002) | **-0.25 (-0.34, -0.16)** | 0.04 (-0.17, 0.25) | -0.17 (-0.37, 0.04) |
| Trabecular vBMD | **0.09 (0.01, 0.18)** | **-0.10 (-0.19, -0.0003)** | **-0.28 (-0.36, -0.20)** | -0.03 (-0.20, 0.14) | **-0.31 (-0.49, -0.13)** |
| **Cortical bone microarchitecture** |  |  |  |  |  |
| Cortical thickness | **0.29 (0.19, 0.38)** | -0.03 (-0.15, 0.08) | **-0.23 (-0.35, -0.10)** | 0.20 (-0.11, 0.51) | 0.14 (-0.12, 0.40) |
| Cortical perimeter | **0.26 (0.17, 0.35)** | **0.04 (0.02, 0.06)** | **0.12 (0.09, 0.15)** | **0.08 (0.05, 0.11)** | **0.26 (0.21, 0.31)** |
| **Trabecular microarchitecture** |  |  |  |  |  |
| Tb.BV/TV^d^ | **0.10 (0.01, 0.18)** | **-0.10 (-0.19, -0.0006)** | **-0.28 (-0.36, -0.20)** | -0.03 (-0.20, 0.14) | **-0.31 (-0.49, -0.13)** |
| Trabecular number | 0.01 (-0.16, 0.18) | **-0.23 (-0.36, -0.10)** | **-0.29 (-0.44, -0.14)** | **-0.36 (-0.56, -0.17)** | **-0.45 (-0.68, -0.21)** |
| Trabecular thickness | **0.11 (0.05, 0.17)** | 0.06 (-0.05, 0.17) | -0.07 (-0.18, 0.05) | **0.26 (0.10, 0.41)** | 0.00 (-0.18, 0.18) |
| Trabecular separation | 0.05 (-0.14, 0.24) | **0.26 (0.12, 0.40)** | **0.30 (0.17, 0.44)** | **0.28 (0.14, 0.43)** | **0.40 (0.23, 0.56)** |
| Tb.1/N.SD^d^ | 0.17 (-0.08, 0.41) | **0.17 (0.06, 0.29)** | **0.18 (0.06, 0.30)** | **0.32 (0.21, 0.43)** | **0.30 (0.16, 0.44)** |

Beta coefficients represent a 1 unit increase in osteophyte score per SD change in HRpQCT measure.

*Multivariable linear regression adjusting for age, sex, and BMI.

^d^ parameters were calculated using the derived measurement method.

Bold denotes statistical significance.

Abbreviations: SD: standard deviation; CI: confidence interval; CMC: carpometacarpal joint; DIP: distal interphalangeal joint; PIP: proximal interphalangeal joint; vBMD: volumetric bone density, Tb.BV/TV: Trabecular bone volume fraction, Tb.1/N.SD: Inhomogeneity of trabecular network.
