# Supplementary material for: Association between radiographic hand osteoarthritis and bone microarchitecture in a population-based sample
Source: Arthritis Res Ther. 2022 Sep 17;24:223. doi: 10.1186/s13075-022-02907-6 (PMC9482179; doi:10.1186/s13075-022-02907-6)
Supplement: Supplementary file 5 — Additional file 5: Supplementary Table 4. Standardized beta-coefficients for the associations of site-specific osteophyte and joint space narrowing scores with HRpQCT measures (per SD) stratified by 1st CMC, distal and proximal sites (N=201) further adjustment for alcohol intake, current smoking, physical activity, occupational impact. [file 13075_2022_2907_MOESM5_ESM.docx]

**Supplementary Table 4.** Standardized beta-coefficients for the associations of site-specific osteophyte and joint space narrowing scores with HRpQCT measures (per SD) stratified by 1st CMC, distal and proximal sites (N=201) further adjustment for alcohol intake, current smoking, physical activity, occupational impact

|  | Osteophyte scores | | |  | Joint space narrowing scores | | | |
| --- | --- | --- | --- | --- | --- | --- | --- | --- |
|  | 1^st^ CMC  β (95% CI)* | Distal site  β (95% CI)† | Proximal site  β (95% CI)† |  | 1^st^ CMC  β (95% CI)* | Distal site  β (95% CI)† | | Proximal site  β (95% CI)† |
| **Areas and density** |  |  |  |  | | |  |  |
| Total bone area | **0.33 (0.24, 0.43)** | **0.08 (0.05, 0.11)** | **0.22 (0.18, 0.26)** | **0.38 (0.27, 0.48)** | | | **0.03 (0.01, 0.05)** | **0.09 (0.08, 0.11)** |
| Cortical area | **0.56 (0.39, 0.73)** | **0.19 (0.05, 0.34)** | **0.17 (0.01, 0.33)** | **0.66 (0.48, 0.83)** | | | 0.10 (0.02, 0.18) | 0.06 (-0.01, 0.13) |
| Trabecular area | **0.35 (0.25, 0.45)** | **0.06 (0.02, 0.11)** | **0.22 (0.16, 0.28)** | **0.38 (0.27, 0.48)** | | | 0.01 (-0.01, 0.04) | **0.09 (0.06, 0.12)** |
| Total vBMD | **0.07 (0.004, 0.14)** | -0.002 (-0.17, 0.17) | **-0.52 (-0.74, -0.30)** | **0.09 (0.01, 0.16)** | | | 0.01 (-0.08, 0.11) | **-0.23 (-0.33, -0.13)** |
| Cortical vBMD | 0.09 (-0.02, 0.19) | 0.01 (-0.16, 0.19) | **-0.54 (-0.75, -0.33)** | 0.09 (-0.02, 0.19) | | | -0.02 (-0.11, 0.08) | **-0.22 (-0.31, -0.12)** |
| Trabecular vBMD | 0.08 (-0.004, 0.16) | -0.13 (-0.27, 0.02) | **-0.55 (-0.73, -0.37)** | **0.10 (0.02, 0.19)** | | | -0.07 (-0.15, 0.01) | **-0.26 (-0.34, -0.18)** |
| **Cortical bone microarchitecture** |  |  |  |  | | |  |  |
| Cortical thickness | **0.24 (0.15, 0.33)** | 0.16 (-0.06, 0.38) | -0.17 (-0.44, 0.10) | **0.29 (0.20, 0.39)** | | | 0.07 (-0.05, 0.20) | -0.11 (-0.23, 0.01) |
| Cortical perimeter | **0.27 (0.18, 0.35)** | **0.10 (0.07, 0.13)** | **0.34 (0.28, 0.40)** | **0.26 (0.17, 0.35)** | | | **0.05 (0.04, 0.07)** | **0.16 (0.13, 0.19)** |
| **Trabecular microarchitecture** |  |  |  |  | | |  |  |
| Tb.BV/TV^d^ | 0.08 (-0.001, 0.17) | -0.13 (-0.28, 0.02) | **-0.55 (-0.73, -0.37)** | **0.11 (0.02, 0.19)** | | | -0.07 (-0.15, 0.01) | **-0.26 (-0.34, -0.18)** |
| Trabecular number | -0.0008 (-0.16, 0.15) | **-0.28 (-0.47, -0.08)** | **-0.51 (-0.80, -0.21)** | 0.02 (-0.16, 0.19) | | | **-0.29 (-0.39, -0.18)** | **-0.33 (-0.46, -0.21)** |
| Trabecular thickness | **0.10 (0.04, 0.16)** | 0.11 (-0.04, 0.25) | -0.14 (-0.37, 0.08) | **0.12 (0.06, 0.18)** | | | **0.15 (0.07, 0.23)** | -0.02 (-0.12, 0.08) |
| Trabecular separation | 0.07 (-0.10, 0.24) | **0.26 (0.06, 0.46)** | **0.58 (0.33, 0.83)** | 0.07 (-0.11, 0.24) | | | **0.29 (0.19, 0.40)** | **0.33 (0.22, 0.43)** |
| Tb.1/N.SD^d^ | **0.22 (0.01, 0.43)** | **0.29 (0.15, 0.44)** | **0.43 (0.21, 0.65)** | 0.20 (-0.02, 0.42) | | | **0.24 (0.16, 0.32)** | **0.21 (0.11, 0.31)** |

Beta coefficients represent a 1 unit increase in osteophyte/JSN score per SD change in HRpQCT measure.

*Multivariable linear regression adjusting for age, sex, and BMI, alcohol intake, current smoking, physical activity, occupational impact.

†Mixed-effects model including fixed effects for age, sex, BMI, alcohol intake, current smoking, physical activity, occupational impact and random intercepts for ROIs.

^d^ parameters were calculated using the derived measurement method.

Bold denotes statistical significance.

Distal site: distal 2^nd^ distal interphalangeal joint, distal 2^nd^ proximal interphalangeal joint. Proximal site: proximal 2^nd^ distal interphalangeal joint, proximal 2^nd^ proximal interphalangeal joint.

Abbreviations: SD: standard deviation; CI: confidence interval; CMC: carpometacarpal joint; vBMD: volumetric bone density, Tb.BV/TV: Trabecular bone volume fraction, Tb.1/N.SD: Inhomogeneity of trabecular network.
