# Supplementary material for: Association between radiographic hand osteoarthritis and bone microarchitecture in a population-based sample
Source: Arthritis Res Ther. 2022 Sep 17;24:223. doi: 10.1186/s13075-022-02907-6 (PMC9482179; doi:10.1186/s13075-022-02907-6)
Supplement: Supplementary file 6 — Additional file 6: Supplementary Table 5. Standardized beta-coefficients for the associations of site-specific osteophyte and joint space narrowing scores with HRpQCT measures (per SD) stratified by 1st CMC, distal and proximal sites (N=201) further adjusted for self-reported diagnosis of osteoporosis. [file 13075_2022_2907_MOESM6_ESM.docx]

**Supplementary Table 5:** Standardized beta-coefficients for the associations of site-specific osteophyte and joint space narrowing scores with HRpQCT measures (per SD) stratified by 1st CMC, distal and proximal sites (N=201) further adjusted for self-reported diagnosis of osteoporosis

|  | Osteophyte scores | | |  | Joint space narrowing scores | | | |
| --- | --- | --- | --- | --- | --- | --- | --- | --- |
|  | 1^st^ CMC  β (95% CI)* | Distal site  β (95% CI)† | Proximal site  β (95% CI)† |  | 1^st^ CMC  β (95% CI)* | Distal site  β (95% CI)† | | Proximal site  β (95% CI)† |
| **Areas and density** |  |  |  |  | | |  |  |
| Total bone area | **0.35 (0.26, 0.44)** | **0.07 (0.05, 0.10)** | **0.20 (0.17, 0.24)** | **0.38 (0.28, 0.48)** | | | **0.03 (0.01, 0.04)** | **0.08 (0.07, 0.10)** |
| Cortical area | **0.54 (0.38, 0.71)** | **0.14 (0.01, 0.28)** | **0.17 (0.01, 0.32)** | **0.64 (0.47, 0.82)** | | | 0.07 (-0.01, 0.14) | 0.04 (-0.03, 0.10) |
| Trabecular area | **0.35 (0.26, 0.45)** | **0.06 (0.02, 0.10)** | **0.20 (0.14, 0.25)** | **0.37 (0.26, 0.47)** | | | 0.01 (-0.01, 0.04) | **0.08 (0.06, 0.11)** |
| Total vBMD | 0.06 (-0.01, 0.13) | -0.03 (-0.19, 0.12) | **-0.47 (-0.68, -0.27)** | **0.08 (0.01, 0.15)** | | | -0.03 (-0.12, 0.06) | **-0.26 (-0.35, -0.16)** |
| Cortical vBMD | 0.07 (-0.02, 0.17) | -0.04 (-0.20, 0.12) | **-0.48 (-0.68, -0.29)** | 0.06 (-0.04, 0.17) | | | -0.05 (-0.14, 0.04) | **-0.24 (-0.32, -0.15)** |
| Trabecular vBMD | 0.06 (-0.02, 0.14) | -0.10 (-0.24, 0.03) | **-0.53 (-0.70, -0.36)** | **0.09 (0.01, 0.18)** | | | **-0.10 (-0.18, -0.02)** | **-0.28 (-0.35, -0.21)** |
| **Cortical bone microarchitecture** |  |  |  |  | | |  |  |
| Cortical thickness | **0.22 (0.14, 0.31)** | 0.09 (-0.11, 0.30) | -0.16 (-0.42, 0.10) | **0.28 (0.19, 0.38)** | | | 0.02 (-0.10, 0.14) | **-0.15 (-0.26, -0.03)** |
| Cortical perimeter | **0.26 (0.19, 0.34)** | **0.09 (0.07, 0.12)** | **0.31 (0.26, 0.36)** | **0.26 (0.17, 0.35)** | | | **0.04 (0.03, 0.06)** | **0.15 (0.12, 0.17)** |
| **Trabecular microarchitecture** |  |  |  |  | | |  |  |
| Tb.BV/TV^d^ | 0.07 (-0.01, 0.15) | -0.10 (-0.24, 0.03) | **-0.53 (-0.70, -0.36)** | **0.10 (0.01, 0.18)** | | | **-0.10 (-0.18, -0.02)** | **-0.28 (-0.35, -0.21)** |
| Trabecular number | -0.03 (-0.19, 0.12) | **-0.27 (-0.45, -0.08)** | **-0.59 (-0.86, -0.32)** | 0.01 (-0.16, 0.18) | | | **-0.28 (-0.38, -0.18)** | **-0.35 (-0.47, -0.23)** |
| Trabecular thickness | **0.10 (0.04, 0.16)** | 0.11 (-0.03, 0.25) | -0.04 (-0.25, 0.17) | **0.11 (0.05, 0.17)** | | | **0.11 (0.03, 0.19)** | -0.03 (-0.12, 0.06) |
| Trabecular separation | 0.09 (-0.09, 0.26) | **0.23 (0.06, 0.41)** | **0.64 (0.41, 0.87)** | 0.05 (-0.14, 0.24) | | | **0.28 (0.19, 0.38)** | **0.34 (0.23, 0.44)** |
| Tb.1/N.SD^d^ | **0.23 (0.01, 0.45)** | **0.29 (0.15, 0.43)** | **0.46 (0.27, 0.66)** | 0.16 (-0.08, 0.41) | | | **0.21 (0.13, 0.29)** | **0.22 (0.13, 0.31)** |

Beta coefficients represent a 1 unit increase in osteophyte/JSN score per SD change in HRpQCT measure.

*Multivariable linear regression adjusting for age, sex, and BMI, self-reported diagnosis of osteoporosis.

†Mixed-effects model including fixed effects for age, sex, BMI, self-reported diagnosis of osteoporosis, and random intercepts for ROIs.

^d^ parameters were calculated using the derived measurement method.

Bold denotes statistical significance.

Distal site: distal 2^nd^ distal interphalangeal joint, distal 2^nd^ proximal interphalangeal joint. Proximal site: proximal 2^nd^ distal interphalangeal joint, proximal 2^nd^ proximal interphalangeal joint.

Abbreviations: SD: standard deviation; CI: confidence interval; CMC: carpometacarpal joint; vBMD: volumetric bone density, Tb.BV/TV: Trabecular bone volume fraction, Tb.1/N.SD: Inhomogeneity of trabecular network.
