# Supplementary material for: Association between radiographic hand osteoarthritis and bone microarchitecture in a population-based sample
Source: Arthritis Res Ther. 2022 Sep 17;24:223. doi: 10.1186/s13075-022-02907-6 (PMC9482179; doi:10.1186/s13075-022-02907-6)
Supplement: Supplementary file 7 — Additional file 7: Supplementary Table 6. Standardized beta-coefficients for the associations of osteophyte and joint space narrowing scores at hand joints with HRpQCT measures (per SD) at distal radius (N=201). [file 13075_2022_2907_MOESM7_ESM.docx]

**Supplementary Table 6:** Standardized beta-coefficients for the associations of osteophyte and joint space narrowing scores at hand joints with HRpQCT measures (per SD) at distal radius (N=201)

|  | Osteophyte scores | | | | Joint space narrowing scores | | |
| --- | --- | --- | --- | --- | --- | --- | --- |
|  | 1^st^ CMC  β (95% CI)* | Distal site  β (95% CI)† | Proximal site  β (95% CI)† |  | 1^st^ CMC  (95% CI)* | Distal site  β (95% CI)† | Proximal site  β (95% CI)† |
| **Areas and density** |  |  |  | |  |  |  |
| Total bone area | 0.01 (-0.09, 0.12) | -0.07 (-0.20, 0.07) | -0.11 (-0.29, 0.07) | | -0.01 (-0.12, 0.11) | -0.06 (-0.14, 0.01) | -0.06 (-0.14, 0.01) |
| Cortical area | -0.002 (-0.12, 0.12) | 0.08 (-0.07, 0.23) | -0.04 (-0.24, 0.16) | | 0.01 (-0.13, 0.14) | -0.12 (-0.20, 0.06) | -0.09 (-0.20, 0.20) |
| Trabecular area | 0.01 (-0.10, 0.13) | -0.10 (-0.25, 0.05) | -0.12 (-0.32, 0.09) | | -0.01 (-0.14, 0.12) | -0.05 (-0.14, 0.04) | -0.05 (-0.14, 0.04) |
| Total vBMD | 0.03 (-0.12, 0.18) | 0.08 (-0.11, 0.28) | -0.07 (-0.33, 0.19) | | -0.08 (-0.24, 0.08) | -0.07 (-0.19, 0.04) | -0.07 (-0.19, 0.04) |
| Cortical vBMD | -0.03 (-0.18, 0.12) | -0.05 (-0.24, 0.14) | -0.10 (-0.35, 0.15) | | -0.05 (-0.21, 0.11) | -0.04 (-0.19, 0.10) | -0.12 (-0.25, 0.02) |
| Trabecular vBMD | 0.07 (-0.07, 0.21) | 0.04 (-0.14, 0.23) | -0.07 (-0.32, 0.17) | | -0.10 (-0.25, 0.05) | -0.04 (-0.15, 0.07) | -0.04 (-0.15, 0.07) |
| **Cortical bone microarchitecture** |  |  |  | |  |  |  |
| Cortical thickness | -0.01 (-0.15, 0.14) | 0.10 (-0.08, 0.28) | -0.04 (-0.28, 0.21) | | -0.01 (-0.17, 0.14) | -0.09 (-0.22, 0.03) | 0.02 (-0.22, 0.26) |
| Cortical perimeter | 0.04 (-0.08, 0.16) | -0.09 (-0.24, 0.06) | -0.12 (-0.32, 0.08) | | -0.01 (-0.14, 0.11) | -0.07 (-0.15, 0.02) | -0.07 (-0.15, 0.02) |
| **Trabecular microarchitecture** |  |  |  | |  |  |  |
| Tb.BV/TV^d^ | 0.07 (-0.07, 0.21) | 0.04 (-0.14, 0.23) | -0.07 (-0.32, 0.17) | | -0.10 (-0.25, 0.05) | -0.04 (-0.15, 0.07) | -0.04 (-0.15, 0.07) |
| Trabecular number | 0.09 (-0.04, 0.23) | -0.01 (-0.19, 0.18) | -0.06 (-0.31, 0.18) | | -0.09 (-0.23, 0.06) | -0.05 (-0.16, 0.06) | -0.05 (-0.16, 0.06) |
| Trabecular thickness | -0.02 (-0.14, 0.10) | 0.04 (-0.16, 0.25) | -0.11 (-0.38, 0.16) | | -0.07 (-0.19, 0.06) | 0.04 (-0.07, 0.16) | 0.04 (-0.07, 0.16) |
| Trabecular separation | -0.02 (-0.11, 0.06) | -0.01 (-0.21, 0.19) | -0.07 (-0.34, 0.19) | | 0.08 (-0.01, 0.16) | 0.09 (-0.03, 0.20) | 0.09 (-0.03, 0.20) |
| Tb.1/N.SD^d^ | -0.01 (-0.12, 0.09) | 0.01 (-0.19, 0.21) | -0.09 (-0.35, 0.18) | | 0.10 (-0.02, 0.21) | 0.07 (-0.04, 0.19) | 0.07 (-0.04, 0.19) |

*Multivariable linear regression adjusting for age, sex, and BMI, alcohol intake, current smoking, physical activity, occupational impact.

†Mixed-effects model including fixed effects for age, sex, BMI, alcohol intake, current smoking, physical activity, occupational impact and random intercepts for ROIs.

^d^ parameters were calculated using the derived measurement method.

No statistical significances.

Distal site: distal 2^nd^ distal interphalangeal joint, distal 2^nd^ proximal interphalangeal joint. Proximal site: proximal 2^nd^ distal interphalangeal joint, proximal 2^nd^ proximal interphalangeal joint.

Abbreviations: SD: standard deviation; CI: confidence interval; CMC: carpometacarpal joint; vBMD: volumetric bone density, Tb.BV/TV: Trabecular bone volume fraction, Tb.1/N.SD: Inhomogeneity of trabecular network.
